# Incorporating *Ab Initio* energy into threading approaches for protein structure prediction

**DOI:** 10.1186/1471-2105-12-S1-S54

**Published:** 2011-02-15

**Authors:** Mingfu Shao, Sheng Wang, Chao Wang, Xiongying Yuan, Shuai Cheng Li, Weimou Zheng, Dongbo Bu

**Affiliations:** 1 Institute of Computing Technology, Chinese Academy of Sciences, Beijing, China; 2 Institute of Theoretical Physics, Chinese Academy of Sciences, Beijing, China; 3 International Computer Science Institute, Berkeley

## Abstract

**Background:**

Native structures of proteins are formed essentially due to the combining effects of local and distant (in the sense of sequence) interactions among residues. These interaction information are, explicitly or implicitly, encoded into the scoring function in protein structure prediction approaches—threading approaches usually measure an alignment in the sense that how well a sequence adopts an existing structure; while the energy functions in *Ab Initio* methods are designed to measure how likely a conformation is near-native. Encouraging progress has been observed in structure refinement where knowledge-based or physics-based potentials are designed to capture distant interactions. Thus, it is interesting to investigate whether distant interaction information captured by the *Ab Initio* energy function can be used to improve threading, especially for the weakly/distant homologous templates.

**Results:**

In this paper, we investigate the possibility to improve alignment-generating through incorporating distant interaction information into the alignment scoring function in a nontrivial approach. Specifically, the distant interaction information is introduced through employing an *Ab Initio* energy function to evaluate the “partial” decoy built from an alignment. Subsequently, a local search algorithm is utilized to optimize the scoring function.

Experimental results demonstrate that with distant interaction items, the quality of generated alignments are improved on 68 out of 127 query-template pairs in Prosup benchmark. In addition, compared with state-to-art threading methods, our method performs better on alignment accuracy comparison.

**Conclusions:**

Incorporating *Ab Initio* energy functions into threading can greatly improve alignment accuracy.

## Introduction

Protein structure determination is critical for understanding protein functions, and also highly relevant with therapeutics and drugs design. Computational prediction methods for protein structure play important roles due to the speed of experimental determination methods cannot catch up with that of generation of protein primary sequences by genome projects. Computational protein structure prediction methods can be categorized into free modeling (FM) and template-based modeling (TBM). Specifically, for the protein without structural analogs in the template database, the structural conformation has to be built from the scratch; while for the proteins having structural analogs, the key step is to identify an accurate alignment between the query sequence and a template with known structure.

Both *Ab Initio* and threading approaches employ scoring functions to capture interactions among residues in an explicit or implicit manner. In essence, protein folding is the combining effects of local interactions and distant interactions among residues. Specifically, local interactions lead to local structural motifs, while non-local interactions arrange local structural motif to form native-like structures.

The *Ab Initio* approaches for free modeling attempt to find a structural conformation with the lowest energy. Typically, local interactions are described via short structural fragments while nonlocal interactions are captured via an energy function. Various energy functions [1-5] have been proposed, and can be categorized into two classes, i.e., knowledge-based and physics-based. Compared with physics-based energy functions, knowledge-based energy functions are more attractive since they are easy to use and understand. In addition, distance-dependent potentials perform better than distance-independent ones [6].

A typical template-based modeling procedure consists of a threading step to align the target protein onto a template, and a refinement step to refine the template structure to be more native-like. Numerous threading methods have been proposed to calculate the optimal alignments under different scoring functions. These threading methods can be categorized into the following classes based on the divergence of scoring functions:

1. The scoring function does not contain any non-local interaction information explicitly. For example, FASTA [7], BLAST [8], and PSI-BLAST [9] assume independence among residues at different positions while HMMer [10] and HHpred [11] apply Hidden Markov Model to introduce the transition information between adjacent residues into scoring function. Since only local information is taken into consideration in their scoring functions, dynamic programming is a natural technique to obtain a global optimal solution.

2. The scoring function captures non-local interaction information via contact preference. That is, if a pair of residues in the query sequence are aligned to the two ends of an interaction, then this pair will be given a score according to a contact preference matrix. PROSPECT [12] and RAPTOR [13] implemented this kind of energy function and demonstrated the improvements of prediction accuracy. However, the following features of non-local interactions were not taken into consideration explicitly: (i) it is more accurate to describe pairwise interactions in distance-dependent manner than distance-independent ways; and (ii) besides distance, the orientation angles involved in dipole–dipole interactions have also been proved to be useful to discriminate native structures.

The purposes of the study is to investigate whether threading results can be improved through incorporating *Ab Initio* energy function. Distant interactions are usually described in a more accurate manner in *Ab Initio* energy function. For example, dDFIRE [6] employs distance-dependent pair-wise interaction rather than distance-independent one. Encouraging progress has been observed in structure refinement where *Ab Initio* energy function is employed to refine template structure to be more native-like. It is interesting whether *Ab Initio* energy function improves alignment generating.

In addition, when the global structural information is incorporated, effective algorithms such as dynamic programming do not work any more: if all pairwise interactions are added into scoring function, the optimization problem becomes NP-hard [14]. A variety of techniques, such as integer linear programming [13] and divide and conquer [15] have been proposed to solve this problem. In this study, we propose an efficient, local search based method to identify optimal alignments. Comparing with existing methods [13,15], which are designed specifically for scoring functions consisting of distant-independent pairwise interaction alone as their global item, our method is more general and can be used to optimize any kind of scoring functions.

## Scoring model

The scoring function to assess an alignment ***A*** consists of local item *L*(***A***) and distant item *G*(***A***), i.e., *score*(***A***) = *ω_L_L*(***A***) + *G*(***A***), where *ω_L_* denotes weight of local item.

Local item is the weighted sum of mutation score *S_m_*, secondary structure compatibility score *S_ss_*, solvent accessibility score *S_sa_*, gap penalty score *S_g_*, and structural segment compatibility score *S_CLE_*[*16*], i.e., *L*(***A***) = *ω_m_S_m_*(***A***) + *ω_ss_S_ss_*(***A***) + *ω_CLE_S_CLE_*(***A***) + *ω_sa_S_sa_*(***A***) + *ω_g_S_g_*(***A***), *S_g_*(***A***) = *ω_go_GO* + *ω_ge_GE*, where *GO* and *GE* are the number of gap open and gap extending, respectively. The weight of these items are to be determined via training on SALIGN benchmark.

The global item *G*(***A***), which contains the nonlocal interaction information implicitly, is captured by the dDFIRE energy over a “partial” decoy corresponding to the alignment ***A***. An ideal way to measure non-local interaction is to calculate dDFIRE energy over a full-length decoy. However, it is usually time-consuming to obtain full-length decoy through running structure-generating tools such as MODELLER [17]. Thus, this strategy is unacceptable since we usually need to sample thousands of alignments. Here, we employ an alternative method to build a partial “decoy” from the alignment. Specifically, only the aligned residues are kept with their coordinates simply copied from the corresponding residues in the template.

This section are organized as follows: We first verify that dDFIRE energy function is constantly good-performing when used to evaluate “partial” decoys. Second, both local item and global item should be normalized using match state size. Third, we prove that global item of the our scoring function is effective to capture distance interaction comparing with contact-preference based scoring functions. Fourth, we show that optimal local score can be used to determine “easy” pairs for which local score item is sufficient while adding global item may lead noise contrarily. Last, we train *ω_L_* on SALIGN [18] benchmark dataset.

### Performance of dDFIRE on partial structure

Since we calculate dDFIRE energy on the “partial” decoy instead of a full-length structure, thus it is necessary to verify whether the “partial dDFIRE energy” still have the power to distinguish native-like decoys. To verify this, we performed experiments on three commonly-used benchmark datasets: LKF [1], Gapless Threading [2] and Rosetta [5]. The datasets contain 178, 200 and 232 proteins, respectively; and for each protein, 100 decoys were generated as control to the native structure. The objective of this experiment is to verify whether the “partial” native structure can be distinguished from the “partial” decoys by dDFIRE.

**Figure 1:**
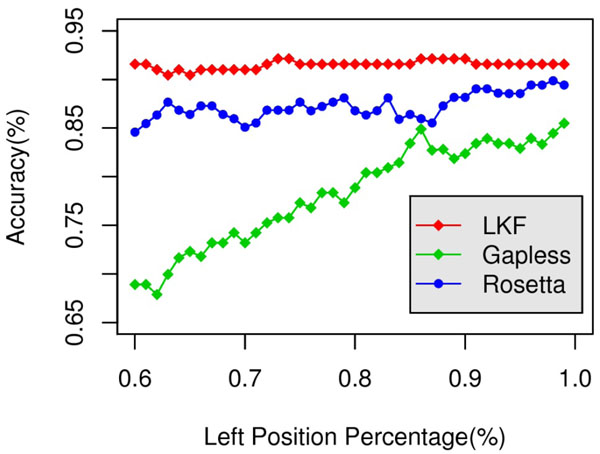
**Performance of dDFIRE to distinguish “partial” native structure from “partial” decoys.** X-axis is the ratio of remaining residues after the excising process, and Y-axis denotes the ratio of proteins for which the “partial” native structure still have lower energy than “partial” decoys.

For both native structures and decoys, the “partial” conformations were simulated through randomly excising a set of residues. At various excising percentage, the ratio of proteins for which the partial native structure has the lowest dDFIRE energy relative to all partial decoys are calculated, and denoted as accuracy in Fig.1. As demonstrated by Fig.1, on LKF and Rosetta benchmarks, dDFIRE performs constantly well even if over 40% residues are excised; and on Gapless Threading benchmark, the performance decreases slightly.

### Score normalization

**Figure 2:**
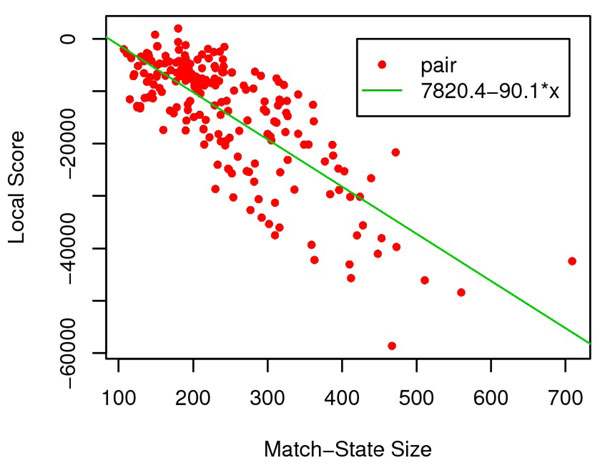
**Linear correlation between local score and match state size.** Both local score and match state size are calculated from reference alignment of query-template pairs in SALIGN [18] benchmark dataset.

**Figure 3:**
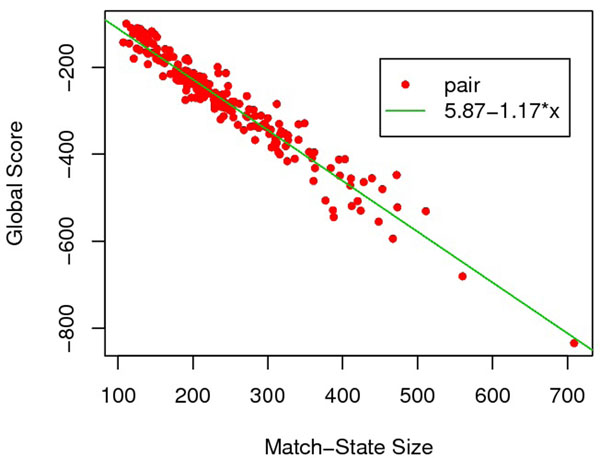
Linear correlation between global score and match state size.

We also investigate the relationship between the scores with the match state size. Analysis suggests the linearity between local(global) scores and match state size. Specifically, the linear correlation coefficient between local(global) scores and the match state size is –0.762 (–0.968) (See Fig.2 and 3 for details). Thus, it is reasonable to normalize both local and global score through dividing by the match state size.

### Effect of global items

We further investigate the effect of global item. As control, we performed comparison with the traditional way to describe non-local interactions via contact preference matrix [13,15], i.e, *S_p_* = ∑*_i_*∑*_j_δ*(*i*, *j*)*Pair*(***A***(*i*), ***A***(*j*)), and  where ***A***(*i*) is the matched residue in the sequence, *δ*(*i*, *j*) indicates whether *i*th and *j*th residue in the template have contact, *P_m_* is the profile vector at the *m*th position and *C* is the contact preference matrix.

We first give some notations before presenting the experiments to examine the effects of global items. For each query-template pair, two typical alignments are generated: the structural alignment ***A****_R_* generated via running TMalign [19], and the optimal alignment (denoted as ***A****_L_*) when only local item *L*(***A***) is taken into consideration, i.e. ***A****_L_* = argmin***_A_****L*(***A***)*.* For each alignment ***A***, its real quality is measured by TMscore [20], denoted as TM(***A***). We also use *L*(***A***) and *G*(***A***) to denote the local score and global score of ***A***, and use *C*(***A***) to denote the contact-preference-based score of ***A***.

**Figure 4:**
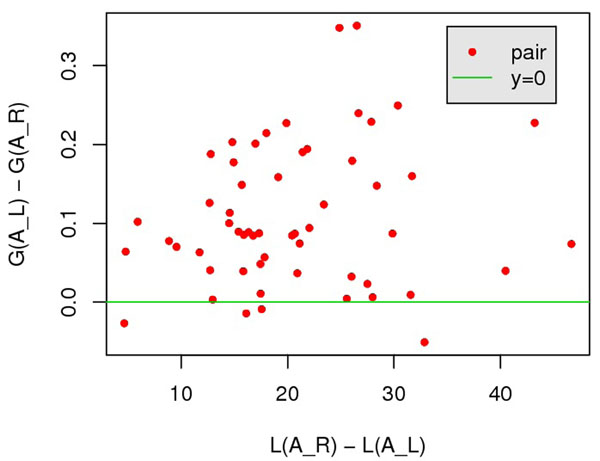
**Effect of global score to distinguish *A_R_* from *A_L_.*** All points lies to the right of *x* = 0, and 52 of 56 points appear above *y* = 0.

**Figure 5:**
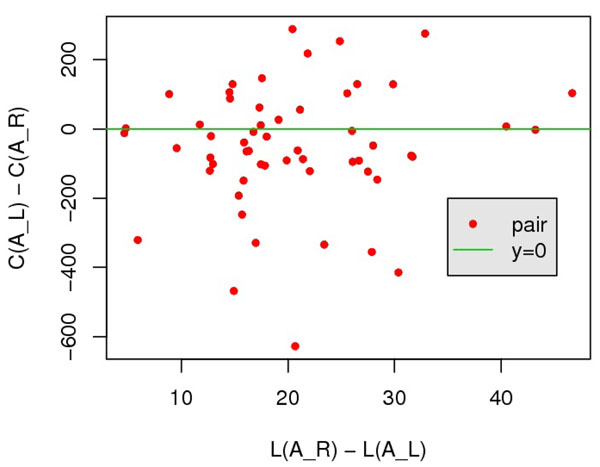
**Effect of contact-preference-based score to distinguish *A_R_* from *A_L_.*** Only 20 of 56 points appear above *y* = 0.

The 200 query-template pairs in SALIGN [18] dataset are categorized into two classes according to the quality of ***A****_L_*: (i) TM(***A***_R_) – TM(***A****_L_*) < 0.1, 144 pairs in total; and (ii) TM(***A****_R_*) – TM(***A****_L_*) ≥ 0.1, 56 pairs in total. Intuitively, class 1 contains the pairs for which a scoring function with local score item alone is sufficient; and class 2 contains the pairs for which local score alone failed. For pairs in class 2, we expect global items can help to distinguish the reference alignment. We verify this by comparing the global score of ***A****_L_* and ***A****_R_* : only for pairs satisfying ***A****_L_* – ***A****_R_* > 0, it is likely to distinguish the reference alignment. Fig.4 and 5 suggest that for the pairs that local item alone cannot separate ***A****_L_* from ***A****_R_* (*L*(***A****_L_*) ≤ *L*(***A****_R_*) because of ***A****_L_* = argmin***_A_****L*(***A***)), global item of our scoring function can effectively measure the quality of alignments. Specifically, we observed that *G*(***A****_R_*) <*G*(***A****_L_*) on 52 of 56 pairs. In contrast, the contact-preference-based score does not help improve this situation, only on 20 of 56 pairs, *C*(***A****_R_*) <*C*(***A****_L_*)*.*

### Determining pairs for which local score item alone is sufficient

On 144 of 200 pairs of SALGIN benchmark, local score alone is sufficient to find out a “good” alignment(TM(***A****_R_*) – TM(***A****_L_*) < 0.1). In fact, in these cases, adding global item may lead to false-negative [21]. We observed that the normalization of local scores help recognizing these “easy” pairs. This is reasonable since local score contains most of the homologous information between the sequence and the template.

**Figure 6:**
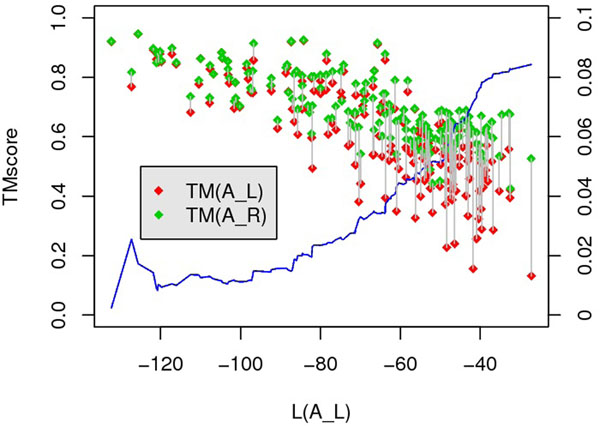
**Linear correlation between TMscore and normalized local score.** For each pair in SALIGN benchmark dataset, TMscore of reference alignment(green points) and TMscore of ***A****_L_* are compared with local score of ***A****_L_.* Length of gray segment represents the difference of TMscore. The average difference of TMscore(using right axis) along with local score increasing is showed as the blue line.

Fig.6 implies that TMscore value is strongly correlated with local score (linear correlation coefficient is -0.78). Besides, as the local score increases, ***A****_L_* becomes worse, i.e., the cumulative average value of TM(***A****_R_*) – TM(***A****_L_*) increases as the local score increasing (the blue curve in Fig.6). Accordingly, we choose a threshold of local score, denoted as *θ*, to determine whether local score item is sufficient: if *L*(***A****_L_*) ≤ *θ*, then ***A****_L_* is treated as a good alignment. In our method, *θ* = –87.

### Weight training process

Parameter *ω_L_* is trained by classification. For each query-template pair in SALIGN benchmark, one positive alignment ***A****_p_* and 10 negative alignments ***A**_n_* are selected (We also have tried other number of negative alignments, similar result is obtained).

Here, we use the reference alignment as positive alignment, i.e. ***A****_p_* = ***A****_R_.* Negative alignments are chosen from the top 100 alignments returned by dynamic programming. We first cluster these alignments to remove redundancy, and then randomly select alignments satisfying TM(***A****_p_*) – TM(***A****_n_*) > 0.2.

*ω_L_* should divide ***A****_n_* and ***A****_p_* as much as possible. Formally

*ω_L_* = arg max *|H|*

where

*H* = {(***A****_p_*, ***A****_n_*) ∈ ***P***|*ω_L_L*(***A****_p_*) + *G*(***A****_p_*) <*ω_L_L*(***A****_n_*) + *G*(***A****_n_*)}

 = {(***A****_p_*, ***A****_n_*) ∈ *P*|*ω_L_L*(***A****_p_*) – *L*(***A****_n_*)) <*G*(***A****_n_*) – *G*(***A****_p_*)}*.*

**Figure 7:**
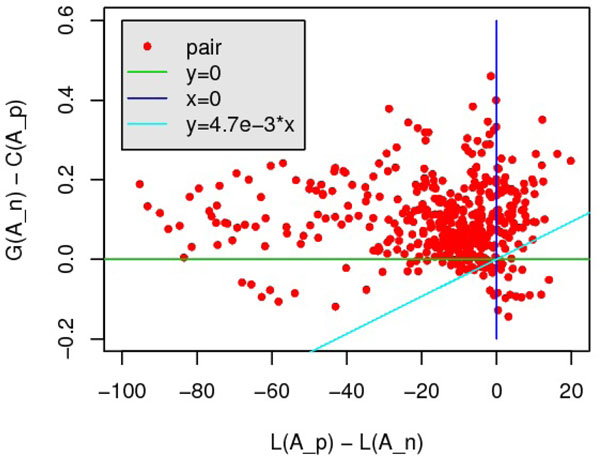
**Training *ω_L_* on SALIGN benchmark.** X-axis is *L*(***A****_p_*) – *L*(***A****_n_*) while Y-axis is *G*(***A****_n_*) – *G*(***A****_p_*)*.* The optimization problem requires a positive-slope line with the most points above it.

***P*** = {(***A****_p_*, ***A****_n_*)*|****A****_p_* and ***A****_n_* are from the same pair}, since alignments of different query-template pair are not comparable. The classification result is showed in Fig.7, *ω_L_* = 0.0047.

After obtaining the parameter *ω_L_* and *θ*, our threading algorithm can be described informally as follows: given a query-template pair, dynamic programming algorithm is employed to calculate ***A****_L_ .* If *L*(***A****_L_*) <*θ*, then ***A****_L_* is considered as a good alignment and returned. Otherwise, local search algorithm is then used to find a better alignment under scoring function *score*(***A***) = *ω_L_L*(***A***) + *G*(***A***)*.* The initial alignments used in this step are chosen from the dynamic programming table in the previous step.

## Preliminary results on alignment generating

We test our threading method on Prosup benchmark (containing 127 query-template pairs). Each query-template pair shares low sequence identity but high structure similarity. Denote the alignment generated by our method as ***A****_O_.*

**Figure 8:**
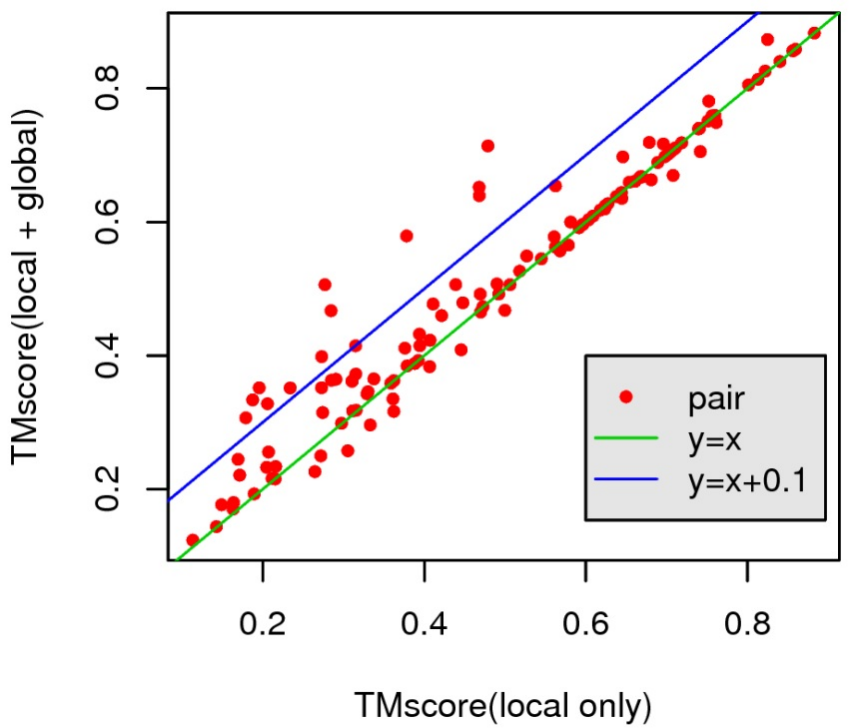
**TMscore comparison between TM(***A****_L_*) and TM(***A****_O_*) on Prosup benchmark.** X-axis is TM(***A****_L_*) while Y-axis is TM(***A****_O_*). Each red point is a pair in Prosup benchmark. Green line is *y* = *x*. Points above this line represents the new scoring function has a better performance. Blue line is *y* = *x* + 0.1. Points above this line represents an improvement over 0.1. 20 points are above blue line.

First, we compare TM(***A****_O_*) with TM(***A****_L_*) in order to evaluate the effect of the new scoring function. The result is showed in Fig.8. It suggests that on 68 out of 127 pairs the new scoring function gains a better TMscore compared with scoring function with local item only. On 12 out of 127 pairs, TMscore improvement is greater than 0.1 while no pair’s TMscore decrease greater than 0.1.

Second, we compare the alignment accuracy with other threading methods. For an alignment, its accurate accuracy is defined as the ratio of number of correct match-state over the number of match-state of the reference alignment; the ratio is denoted as ±4-residues-accuracy if a ±4 error allowed. Experimental results (Table 1) indicate that our method performs better than FASTA, Sequence and PSI-BLAST. If only the local score item is considered, the alignment accuracy is comparable to RAPTOR. When the distant scoring item is added, the alignment accuracy improves significantly: 8% better than RAPTOR on accurate comparison and 6.4% on ±4-residues comparison.

**Table 1:** Alignment Accuracy Comparison on Prosup Benchmark.

Methods	Accurate(%)	±4-residues(%)
FASTA	31.4	-
Sequence	34.1	-
PSI-BLAST	35.6	-
RAPTOR	44.0	63.7
***A*** *L*	43.1	63.0
***A*** *O*	**52.0**	**70.1**

Result of FASTA and Sequence are from [22]; result of PSI-BLAST is from [23]; result of RAPTOR is obtained from the binary version running by us, this result may not reflect the accuracy of the current version.

## Methods

### Threading Algorithm

The framework of our threading algorithm are described as follows:

Algorithm 1(Threading Algorithm)

**Input:** query sequence, template, *θ*, *ω_L_*, *α*, *k*

**Output:** an alignment between query and template

**step 1** set *score*(***A***) = *L*(***A***), calculate the optimal alignment ***A****_L_* under this scoring function by dynamic programming algorithm, save the best 100 alignments from the dynamic programming table

**step 2** calculate *L*(***A****_L_*) , if *L*(***A****_L_*) <*θ*, then return ***A****_L_*

**step 3** set *score*(***A***) = *ω_L_L*(***A***) + *G*(***A***), for each alignment ***A****_i_* in the 100 candidates in step 1, run local search algorithm(Algorithm 2 described in the following subsection) with parameter *α*, *k* and initial alignment ***A****_i_*, it returned ***A****_Oi_*

**step 4** return 

### Local Search Algorithm

In this sub-section we describe the threading problem in a concise way, propose a local search algorithm based on a new neighborhood for general scoring function. Under a certain assumption, we prove its approximation guarantee for two specific scoring functions.

#### Problem Formulation

We first give some formal definitions.

**Definition 1.***Given a template T* = {*t*_1_, *t*_2_, ⋯, *t_m_*}, *t_i_* <*t*_i+1_*and a sequence S* = {*s*_0_, *s*_1_, *s*_2_, ⋯, *s_n_*}, *s_i_* <*s_i_*_+1_, *a valid* alignment *is a non-decreasing mapping****A****from T to S.*

Denote all valid alignments as ***F****.* Non-decreasing mapping is equivalent with traditional alignment definition with *gap.* For all *t* satisfying ***A***(*t*) = *s*, *s* > *s*_0_, we can define the smallest *t* actually matches with *s* while others are gap on template. In order to allow gap on the left end of the template, we add a extra amino acid *s*_0_ in the left end of the sequence. All *t* ∈ *T* aligned to *s*_0_ are gap on the left end of the template. Mapping allows gap on sequence naturally.

Now we define the neighborhood of an alignment. Denote the *k-*neighbor of ***A*** as *N*(***A***, *k*), we have the following definition.

**Definition 2.***Suppose****A***′ ∈ ***F***, *then****A***′ ∈ *N*(***A***, *k*) *if and only if there exists a subset U of S*, *satisfying |U|* ≤ *k and* ∀*t* ∈ *T****A***′(*t*) ∈ {***A***(*t*)} ∪ *U.*

Intuitively, a member of *k*-neighbors of ***A*** differs with ***A*** only on at most *k* positions at sequences.

**Claim 1.***N*(***A***, *k*) ⊂ *N*(***A***, *k* + 1).

**Claim 2.***N*(***A***, *n* + 1) = ***F****.*

**Claim 3.***|N*(***A***, *k*)*|* = *O*(*m*^2^*^k^n^k^*), ∀***A*** ∈ ***F****.*

Claim 1 and claim 2 are obvious. The proof claim 3 is put in the Appendix.

Claim 1 and claim 2 show that with the increasing of *k*, the number of neighbors of an alignment is growing and eventually reaches the whole space. Claim 3 estimates the size of |*N*(***A***, *k*)|. It shows that for a fixed *k* the number of neighbors of a valid alignment is polynomial about *m* and *n*.

**Definition 3.***For any****A*** ∈ ***F***, *there is a real positive number denoted as score*(***A***) *to evaluate****A***, *the* threading problem *is* min***_A_***_∈_***_F_****score*(***A***)*.*

*score*(***A***) is the general representation of scoring function. In this study, *score*(***A***) = *ω_L_L*(***A***) + *G*(***A***)*.*

#### Algorithm

Based on the definition of neighborhood above, we give the local search algorithm as follows:

Algorithm 2(Local Search)

**Input***α* ≥ 0, *k*, initial alignment ***A***_0_

**Output** an approximate local optimal solution of the scoring function *score*(***A***)

**step 1***i* = 0, initialize ***A***_0_ according to input

**step 2** calculate *A_i_*_+1_ = argmin***_A_***_∈_*_N_*_(_***_A_****_i_*_,_*_k_*_)_*score*(***A***)

**step 3** if (1 + *α*)*score*(***A****_i_*_+1_) <*score*(***A****_i_*), *i* = *i* + 1, goto *step 2*

**step 4** output ***A****_i_*

When *α* = 0, we can obtain an accurate local optimal solution. When *α* = 0 and *k* = *n* + 1, we can obtain an accurate global optimal solution.

**Claim 4.***Suppose α* > *0*, *The time complexity of algorithm 2 is O*(*m*^2^*^k^n_k_*log_1+_*_α_M*), *where*

*Proof.* Based on the algorithm, we have

(1 + *α*)^*i*^*score*(***A**_i_*) <*score*(***A***_0_),

which implies that

According to claim 3, each iteration wastes at most *O*(m^2^*^k^n^k^*) time, so the claim is proved.

If a *closing assumption* is satisfied, we can prove two approximation guarantee results when the scoring function only consists of local item and pairwise contact item. Details are listed in the Appendix section.

## Discussion

In order to employ general energy function, the key step is transforming alignment to decoy efficiently In this paper, “partial“ decoy strategy is quick enough but not accurate because only matched residues’ backbone and *C_β_* atoms are kept. Methods that effectively recover other unmatched residues and even side chain atoms according to alignments are imperative.

Though the energy function of *Ab Initio* can be used by threading, the two methods have fundamental difference on the divergence of search space. Actually, the search space of threading is much smaller than that of *Ab Initio* methods because many useful prior knowledge can greatly narrow its search space. For instance, we can restrict that a core on template either totally aligned or totally gaped. This prior has been verified and applied by many threading methods. Consequently, the search space can be reduced to *O*(*N^m^*) where *N* is the number of cores and m the length of query sequence. On average, *N* ≤ 10, it is much smaller than 200^m^, which is the search space of ROSETTA.

In this paper we have proposed a local search algorithm to find out the optimal solution of general scoring function. This algorithm is based on a neighborhood definition, and this neighborhood can also be used by other search strategies such as simulated annealing and genetic algorithm.

## Competing Interests

The authors declare that they have no competing interests.

## Appendix

### Proof of Claim 3

*Proof* There is  cases to choose a sub-set *U* of *S* while *|U|* = *k.* Denote the neighbors of an alignment under a certain *U* as *N_U_*(***A***)*.* So, we only need to prove |*N_U_*(***A***)| = *O*(*m*^2^*^k^*)*.*

We can assume that all positions in *U* are not aligned, that is, there exists no *t* ∈ *T* such that ***A***(*t*) = *u_i_.* If not, say, *u_i_* is aligned, we can extend *S* to  and change  Obviously, |*N_U_*(***A***)| ≤ |*N_U_*_′_(***A***)|.

Consider the sub-problem when *T_i_* = {*t*_1_, *t*_2_, ⋯, *t_i_*}, *U_j_* = {*u*_1_, u_2_, *⋯*, *u_j_*}, 1 ≤ *i* ≤ *m* and 1 ≤ *j* ≤ *k.* For this sub-problem, we define ***A***(*i*, *j*) = {*g* ∈ *N_U_j__* (***A***)*|g*(*t_i_*) = *u_j_*}, and *B*(*i*, *j*) = {***A***′ ∈ *N_U_j__* (***A***)*|A*′(*t_i_*) = *A*(*t_i_*)}*.* Then |*N_U_*(***A***)| = |***A***(*m*, *k*)| + *|B*(*m*, *j*)*|.*

Now we give out the iterative formula. Let *a_i_* = inf{*j*|***A***(*t_j_*) ≥ *u_i_*}. then *a_i_* ≤ *a_i_*_+1_. Without losing generality, in the following prove, we assume that *a_i_* ≤ *a_i_*_+1_. Define *δ*(*x*) = 1 when *x* ≥ 0 and *δ*(*x*) = 0 when *x* < 0, we have

we employ mathematics induction to prove:

|***A***(*i*, *j*)| ≤ *i^j^* if 1 ≤ *i* ≤ *a*_1_

|*A*(*i*, *j*)| ≤ *i^i^*^+j^ if *a*_1_ <*i* <*α_i_*_+1_, 1 ≤ *l* <*j*

|***A***(*i*, *j*)| ≤ *i*^2^*^j^*^–1^ if *i* > *a_j_*

|*B*(*i*, *j*)| ≤ 1 if 1 ≤ *i* <*a*_1_

|*B*(*i*, *j*)| ≤ *i*^2^*^l^*^–1^ if *i* = *a_l_*, 1 ≤ *l* ≤ *j*

|*B*(*i*, *j*)| ≤ *i*^2^*^l^* if *a_l_* <*i* <*α_i_*_+i_, 1 ≤ *l* <*j*

|*B*(*i*, *j*)| ≤ *i^2j^* if *i* > *a_j_.*

Firstly, |*B*(1, *j*)| = |*A*(1, *j*)| = 1. When 1 <*i* <*a*_1_,

When *i* = *a*_1_,

When *a_l_* <*i* <*a_l_*_+1_ ≤ *a_j_*,

Similar deduction can be used in the case of *i* > *a_j_.* So the claim is proved.

### Approximation Guarantee of Local Search Algorithm

In this sub-section, we prove two approximation results under a certain assumption. The neighbor we used here is 1-neighbor. From claim 1 we know that for *k*-neighbor, *k* > 1, we can obtain a better result.

The algorithm’s approximation guarantee is closely linked to the specific form of *score*(***A***)*.* First we only consider *score*(***A***) consists of local items: *score*(***A***) = ∑*_t_*_∈_*_T_m*(*t*, ***A***(*t*))*.* For convenience’s sake, we define the following marks.

Due to the technical reasons, we have to do a assumption. In the process of prove, we only need that local optimal solution and distant optimal solution satisfy the assumption, unfortunately, this does not always hold too.

**Assumption 1.***Given 2 alignments **A** and g, define*

*if****A****_i_* ∈ ***F***, *i* = 0, 1, 2, *⋯*, *n*, *we say****A****and g satisfies closing assumption.*

If the above assumption is satisfied, we have the following theorem.

**Theorem 1.***If score*(***A***) = ∑*_t_*_∈_*_T_ m*(*t*, ***A***(*t*)), ∀***A*** ∈ ***F***, A** is the distant optimal solution, A* is the approximate local optimal solution obtained from algorithm 2 with factor α and k = *1*, A* andA** satisfies closing assumption, *n is the length of given sequence*, *αn* < 1. *then*

*Proof* Define

A* and ***A****_i_* differs only in *T_i_* (in this proof, we abbreviate *Ti*(A**) as *T_i_*), so

*score*(A*) – *score*(***A****_i_*) = *m*(*T_i_*, A*) – *m*(*T_i_*, A**)

From the assumption, we know that ***A****_i_* ∈ ***F***, even more, ***A****_i_* ∈ *N_i_*(A*) ⊂ *N*(A*) which means *score*(A*) ≤ (1 + *α*)*score*(***A****_i_*)*.* Notice that  and *T_i_ ∩ T_j_* = Ø, *i* = *j.* We have,

which implicates the conclusion.

**Corollary 1.***If score*(***A***) = ∑*_t_*_∈_*_T_m*(*t*, ***A***(*t*)), ∀***A*** ∈ ***F***, A* is the accurate local optimal solution, *then score*(A*) = *score*(A**)*.*

*Proof* This is the special case of theorem 1 when *α* = 0.

If pair contact is taken into scoring function: *score*(***A***) = ∑*_t_*_∈_*_T_m*(*t*, ***A***(*t*)) + ∑*_u_*_∈_*_T_*∑*_v_*_∈_*_T_p*(*u*, ***A***(*u*), *v*, ***A***(*v*)) we have following theorem.

**Theorem 2.***If score*(***A***) = ∑*_t_*_∈_*_T_m*(*t*, ***A***(*t*)) + ∑*_u_*_∈_*_T_*∑*_v_*_∈_*_T_p*(*u*, ***A***(*u*), *v*, ***A***(*v*)), *then*

where

*Proof.* Define

then

*score*(A*) – *score*(***A****_i_*)

= *m*(*T_i_*, A*) + *p*(*T_i_*, A*, *T_i_*, A*) + *p*(*T_i_*, A*, *T* – *T_i_*, A*) + *p*(*T* – *T_i_*, A*, *T_i_*, A*) – *m*(*T_i_*, A**) – *p*(*T_i_*, A**, *T_i_*, A**) – *p*(*T_i_*, A**, *T* – *T*_i_, A*) – *p*(*T* – *T_i_*, A*, *T_i_*, A**)

= *m*(*T_i_*, A*) + *p*(*T_i_*, A*, *T_i_*, A*) + 2*p*(*T_i_*, A*, *T* – *T_i_*, A*) – *m*(*T_i_*, A**) – *p*(*T_i_*, A**, *T_i_*, A**) – 2*p*(*T_i_*, A**, *T* – *T_i_*, A*)*.*

*score*(A*) – *score*(***A****_i_*) – *α score*(***A****_i_*)

= *m*(*T_i_*, A*) + *p*(*T_i_*, A*, *T_i_*, A*) + 2*p*(*T_i_*, A*, *T* – *T_i_*, A*) – (1 + *α*)[*m*(*T_i_*, A**) + *p*(*T_i_*, A**, *T_i_*, A**) + 2*p*(*T_i_*, A**, *T* – *T_i_*, A*)] – *α*[*m*(*T* – *T_i_*, A*) + *p*(*T* – *T_i_*, A*, *T* – *T_i_*, A*)] ≤ 0

Move positive items to the left side and negative items to the right side, and sum up with *i* = 0, 1, 2, ⋯, *n*, we have, the left side

the right side

By adjusting the inequality of *L* ≤ *R*, we can obtain the conclusion.
